# Exploring Genetic Data Across Individuals: Design and Evaluation of a Novel Comparative Report Tool

**DOI:** 10.2196/10297

**Published:** 2018-09-24

**Authors:** Lauren Westendorf, Orit Shaer, Christina Pollalis, Clarissa Verish, Oded Nov, Mad Price Ball

**Affiliations:** 1 Human-Computer Interaction Lab Computer Science Wellesley College Wellesley, MA United States; 2 Department of Technology Management & Innovation New York University Tandon School of Engineering Brooklyn, NY United States; 3 Open Humans Foundation Boston, MA United States

**Keywords:** genomics, consumer health informatics

## Abstract

**Background:**

The growth in the availability of personal genomic data to nonexperts poses multiple challenges to human-computer interaction research; data are highly sensitive, complex, and have health implications for individuals and families. However, there has been little research on how nonexpert users explore their genomic data.

**Objective:**

We focus on how to support nonexperts in exploring and comparing their own personal genomic report with those of other people. We designed and evaluated CrossGenomics, a novel tool for comparing personal genetic reports, which enables exploration of shared and unshared genetic variants. Focusing on communicating comparative impact, rarity, and certainty, we evaluated alternative novel interactive prototypes.

**Methods:**

We conducted 3 user studies. The first focuses on assessing the usability and understandability of a prototype that facilitates the comparison of reports from 2 family members. Following a design iteration, we studied how various prototypes support the comparison of genetic reports of a 4-person family. Finally, we evaluated the needs of early adopters—people who share their genetic reports publicly for comparing their genetic reports with that of others.

**Results:**

In the first study, sunburst- and Venn-based comparisons of two genomes led to significantly higher domain comprehension, compared with the linear comparison and with the commonly used tabular format. However, results show gaps between objective and subjective comprehension, as sunburst users reported significantly lower perceived understanding and higher levels of confusion than the users of the tabular report. In the second study, users who were allowed to switch between the different comparison views presented higher comprehension levels, as well as more complex reasoning than users who were limited to a single comparison view. In the third study, 35% (17/49) reported learning something new from comparing their own data with another person’s data. Users indicated that filtering and toggling between comparison views were the most useful features.

**Conclusions:**

Our findings (1) highlight features and visualizations that show strengths in facilitating user comprehension of genomic data, (2) demonstrate the value of affording users the flexibility to examine the same report using multiple views, and (3) emphasize users’ needs in comparison of genomic data. We conclude with design implications for engaging nonexperts with complex multidimensional genomic data.

## Introduction

### Overview

Recent years have seen a sharp increase in the availability of personal genomic data to nonexpert consumers. People with no formal training in genetics can get access to their genomic information by sending a saliva sample to a direct-to-consumer genetic testing provider, and results are delivered using a Web app. Users then must interpret large amounts of complex data involving sensitive issues such as disease risk and carrier status. The interpretation of the data may impact lifestyle decisions, emotional state, and well-being of users and their biological family members.

The availability of extant and complex data in need of understanding by nonexperts is an opportunity for human-computer interaction (HCI) research [1]. Identifying user needs and developing novel ways to help users understand their personal genomic data can make a substantial impact on the well-being of people. However, to date, HCI research on interaction with personal genomic data is in its infancy. Studies that investigated the information practices of personal genomic data users found that nonexperts seek to contextualize and compare their personal data with others (eg, family members and others with similar medical conditions) [2,3]. The family-relevant nature of genetic data highlights the need for tools to enable nonexperts to explore not only their own data but also to compare and contrast it with the data of others.

We present CrossGenomics, the first tool to date for nonexpert engagement with multiple gene variant reports. The tool facilitates the exploration of shared genomic information among family members or a comparison of genomic information with others. Such comparisons enable users to explore what variants they share with others and what sets them apart, thus increasing the understanding of their genetic makeup and enriching the genomic narrative people can construct. There has been a growing interest in exploring how people form social ties around health conditions caused or influenced by genetic characteristics—what Kuznetsov et al have called *biosociality* [2]. In this paper, we discuss findings from 2 user studies focusing on assessing the usability and understandability of comparing genetic reports of family members using alternative prototypes of CrossGenomics. In the third study, we explore how people engage with genomic information of famous people, thereby drawing on the public availability of personal genomic information of a few known people, the growing interest in biosociality, and the trend of self-comparison with celebrities, which is increasingly evident in popular culture [4,5].

Beyond the domain of personal genomics, this study expands on a growing body of work, which identifies needs and opportunities to design information-tracking tools for collaborative monitoring of and reflection on family health [4,6-8]. In particular, the paper makes the following contributions: (1) presenting the iterative design and evaluation for a tool that facilitates multidimensional and multiperson comparisons of complex personal data, (2) analyzing the differences between objective comprehension and perceived understanding and shedding light on discrepancies between subjective and objective knowledge, and (3) exploring user needs in the context comparing personal genetic data across individuals.

### Background

#### Nonexpert Engagement With Personal Informatics

Our work on communicating personal genomics to nonexperts draws upon an increasing body of work in personal informatics, which investigates how to make personal data more understandable for nonexpert users and more *embedded* in everyday lives of people. Rooksby et al proposed the term *Lived Informatics* to highlight that collecting and using personal information is embedded in day-to-day lives of people [9]. A widely accepted model of how people use personal informatics tools is the 5-stage model of Li et al [10], which describes the iterative transition between preparation, collection, integration, reflection, and action. This model had been extended by differentiating stages of reflection [11,12] and characterizing challenges in lived informatics for diverse goals of users [13].

Researchers have also studied design interventions and guidelines for making personal data more understandable and accessible for nonexperts. For example, Rapp and Cena investigated usage patterns, information needs, and challenges of naïve users or those who are new to personal informatics. Their findings highlight important differences compared with experienced users, including reduced tolerance to practical difficulties, ambiguous representations, and unintuitive interaction modalities [14]. They also proposed design strategies to address these issues [14] and studied the impact of a new personal informatics system designed to address the needs of naïve users [15]. Epstein et al visualized subsets of collected location and physical activity data using a variety of presentations [16]. Bentley et al developed a system that aggregates multiple aspects of personal well-being data and provides people with insights based on complex relations using natural language [17]. They showed that users were able to understand complex relationships and change their behavior to improve their well-being.

Designing for nonexpert engagement with personal genomics shares goals with personal informatics. However, there are some important aspects unique to personal genomics, including the dynamic nature of its interpretation (as new scientific discoveries are made), and the varying levels of certainty of evidence regarding the implications of the data for well-being of a person. In addition, although personal genomic data are deeply personal and sensitive, they are shared across biological family members and communities. In this study, we focus on the shared aspect of the data, allowing individuals to compare their genomic information with relevant others.

#### Nonexpert Engagement With Personal Genomic Information

Direct nonexpert user engagement with personal genomic information has been relatively understudied in the HCI field [18]. Several studies investigated the motivation for and subjective experience of genetic testing and of using interactive tools to understand results [19,20].

Leighton et al [19] found that nonexperts misinterpret genetic testing results without appropriate assistance. Kuznetsov et al [2] presented users with their own 23andMe data to understand how they make sense of and contextualize their results. Shaer et al [3] studied how nonexperts who participate in the Personal Genome Project explore their own data. They found that similar to other types of personal information [9], engaging with personal genomics could serve a goal, such as finding information about family ancestry and medical history, or be driven by more general curiosity. Their findings also indicate that nonexperts seek to understand their personal genomic information in the context of other individuals, ancestry information, and family medical history.

Exploring the impact of specific report designs, Haga et al [21] studied alternative formats for a text-based genetic laboratory report. Shaer et al [3] compared alternative designs of visual genetic variant reports and showed that a bubble-chart visualization is more effective than other design approaches. On the basis of this finding, they developed a visual tool for nonexperts to explore their own personal genomics information [22]. In this study, we draw on design recommendations introduced by Shaer et al [22] and apply them to the design of a multiperson genetic report that supports the comparison of multiple genomes.

#### Sharing and Exploring Health Data

In recent years, we have witnessed the rise of cocuration in health and medical contexts by nonexperts [23]. Websites such as TuDiabetes, PatientsLikeMe, and Eat.ly help users to make sense of their experiences and conditions by presenting, sharing, and commenting on health knowledge [24-26]. These websites can elicit new concepts for nonexpert health care vocabularies, coding sets, and classifications [25]. In a first effort to facilitate shared genomic data exploration among biological family members by nonexperts, we designed and evaluated CrossGenomics, which enables users to explore what variants they have in common with others.

#### Comparing Genetic Information

Visual tools that compare personal genomic data of different people were introduced by industry with nonexperts, but such tools focus on ancestry exploration. A visualization offered by 23andMe presents shared chromosomal segments between individuals but does not enable comparison and exploration of health and trait information. Several visual tools are available for comparing multiple genomes, including Ensembl [27], IGV [28], Gitools [29], Circos [30], Genome Data Viewer [31], and OMICtools [32]. However, these tools were designed for *expert* users seeking to discover new genetic associations with traits and disease. Such tools provide access to a large variety of metrics, filters, and visualizations, creating numerous leads for discovery. In contrast, CrossGenomics is intended for nonexperts as a report on the existing state of knowledge with regard to the known effects of genetic variants (through published research). We aim to facilitate nonexpert engagement with the data through exploration, while at the same time communicating the multiple dimensionality and uncertainty inherent to genetic data.

#### Visual Tools for Comparing Complex Datasets

In other domains, tools were developed for users to compare and contrast multidimensional and complex datasets [33-37]. The LifeLines system [38], which displays personal history information, was found to perform better compared with a tabular representation. In the context of energy consumption, Valkanova et al [39] presented an interactive visualization system that compares individuals and communities. Most relevant to our research context are tools that allow comparison between individuals who are related to one another. For instance, to support collaborative exploration of family-related information, Zimmerman et al explored the value of technology-supported parents-teens interactions around issues of finance [40]. These tools demonstrate the potential and need to further consider design guidelines for developing tools for nonexperts to compare complex data.

#### Open Humans

Open Humans [41] is a platform dedicated to enabling individuals to manage data and contribute it to research. It was developed with grants from the Robert Wood Johnson Foundation and the Knight Foundation and is currently supported through grants from the Shuttleworth Foundation. Open Humans enables its volunteers to connect data from a variety of current -omic sources. Individuals can join studies on the site, share data with those studies, and contribute to new research. Open Humans acts as an aggregator of participants and data, enables these participants to join new studies, makes data available via application programming interfaces (APIs), and has features for study recruitment and deployment. Open Humans currently supports a variety of -omic data, including genome and exome data (Harvard Personal Genome Project, Gencove, VCF file donation), genotyping data (23andMe, Ancestry DNA), and microbiome data (American Gut, uBiome). This aggregation of participants and data by Open Humans allowed for the creation and promotion of our study to a pool of potential participants with publicly shared genomic data.

## Methods

### Design

We designed novel interactive gene variant reports that allow nonexpert users to compare variants across individuals. Through an iterative design process, we developed 3 alternative designs of an interactive visual personal genomics comparison tool called CrossGenomics that enables nonexpert users to compare their gene variants with others. We used the data and interpretation created by the GET-Evidence gene variant report [42], which contains a list of gene variants known to be associated with traits or medical conditions. Thus, our interactive reports only present gene variants with known (ie, published) effects.

We focused on highlighting 3 dimensions of the data, which were found to be particularly important when exploring personal genomic information [22]: *impact*, which refers to type of effect (eg, whether the gene variant causes or protects against disease), *rarity*, which highlights gene variants that are especially unusual (high rarity indicates low frequency in the population), and *certainty*, which describes the strength of scientific evidence supporting a putative effect. Comparing and contrasting people’s data across these dimensions can help them explore commonalities and differences, as well as implications for potential future health conditions.

In visualizing these dimensions, we drew upon GenomiX [22], an interactive gene variant report for a single genome, where color, size, and position represent impact, rarity, and certainty, respectively. The 3 visual prototypes we developed for CrossGenomics use the same visual encoding as Genomix for representing individual variants but are varied in the visualization technique used for depicting and comparing multiple gene variant reports.

Across the 3 alternative visual prototypes we created for CrossGenomics, color and size retain the same meaning. Color represents impact—pathogenic (red), benign (gray), protective (blue), pharmacogenetic (purple), or carrier (colored polka dot pattern). These impact categories were derived from industry-standard classifications in GET-Evidence [42]. It is common for published effects to be classified in this manner. Similar classifications are used by ClinVar of National Center for Biotechnology Information [43]. Only variants with known effects are represented. Size represents rarity—the larger the representation of the variants, the rarer the gene variant is.

Users could filter gene variants based on person, health category, potential impact, and certainty of evidence with the options “Well-Established,” “Likely,” and “Uncertain.” These categories of certainty of evidence were determined by the GET-Evidence [42] interpretation. Users could also click on a variant to learn more about it. Information, including the variant’s name, clinical importance, potential effect, and an effect summary, would appear in the sidebar. There was also a clickable glossary to explain scientific jargon.

To represent and facilitate comparisons across multiple genomes, we developed 3 alternative prototypes of CrossGenomics, each based on visualization techniques currently used by experts for comparative genomic data: linear [44] and circular alignments [27,29-31,45] and a Venn diagram [28]. However, as our tool is aimed at nonexperts, in our design, we carefully balanced simplifying the visualizations with highlighting important dimensions and facilitating free exploration.

We iterated on the design of these 3 visual reports by testing prototypes in increasing fidelity with nonexpert users (Mechanical Turk workers), experienced users (nonexperts with access to their personal genomics reports), and genomics domain experts. In each iteration, we refined the design, addressing issues such as ordering, synchronized selection, and filtering and sorting variants, size, and alignment.

In addition, we developed a table-based report that is modeled after the GET-Evidence report [42], to reflect the current report type the users of personal genomics often have access to. We implemented CrossGenomics as a Web app using JavaScript with D3.js.

### CrossGenomics 1.0

**Figure 1:**
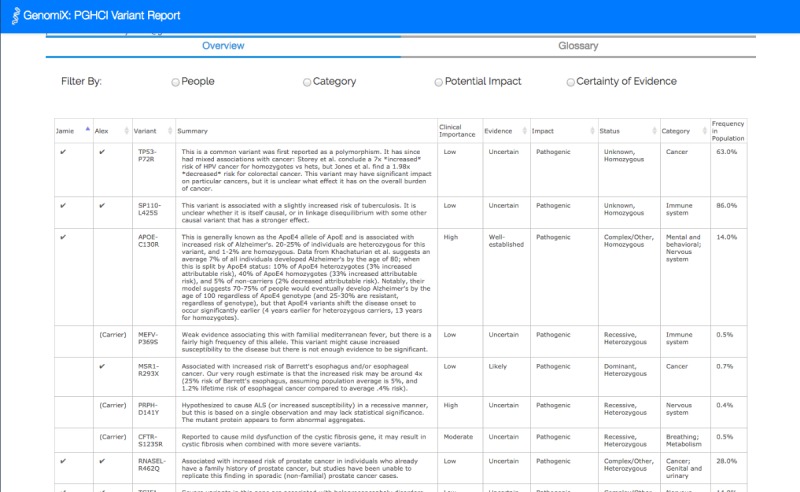
Tabular report: the table is similar to the existing GET-Evidence report with each row representing a variant in one or both of the reports. Two columns were added to the table and check marks were used to denote the presence of a gene variant for each sibling.

**Figure 2:**
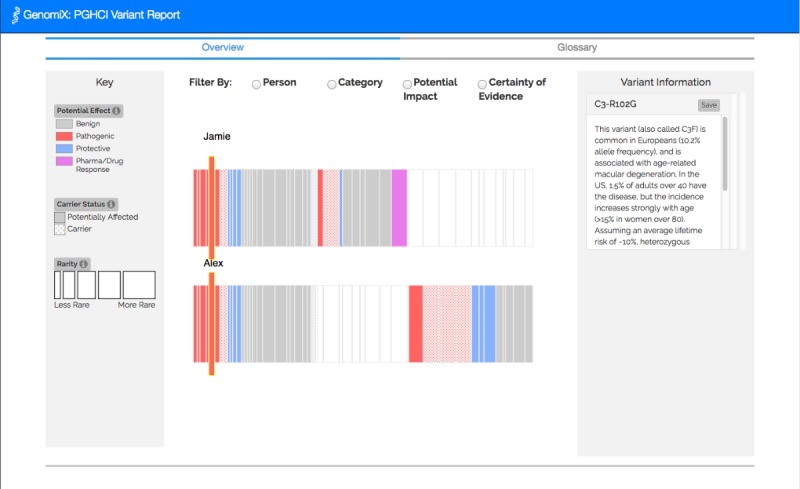
Linear visualization: each rectangle represents a gene variant. Jamie’s variants are represented along the top, Alex’s variants are represented along the bottom, and their shared variants grouped to the left.

**Figure 3:**
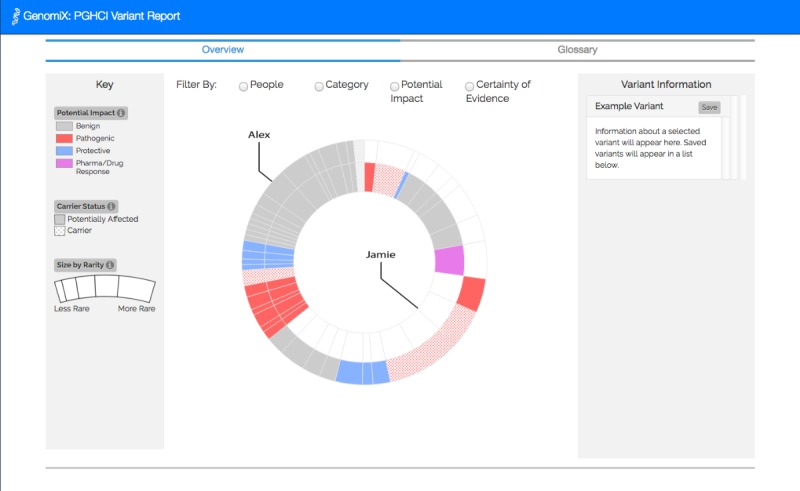
Sunburst visualization: each arc represents a gene variant. The inner circle represents Jamie’s variants and the outer circle represents Alex’s. Variants in both circles represent the ones Jamie and Alex have in common.

**Figure 4:**
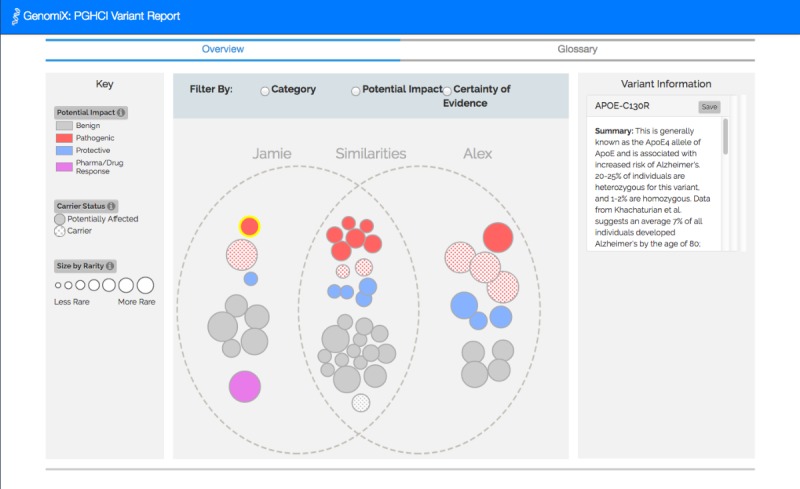
Venn visualization: displays a Venn diagram of gene variants. The bubbles on the left represent Jamie’s variants and bubbles on the right are Alex’s. Bubbles in the middle represent the variants Jamie and Alex have in common.

Figures 1-4 show the 4 alternative designs we developed for comparing personal genetic reports: a table-based report (Figure 1), a linear visualization (Figure 2), a sunburst visualization (Figure 3), and a Venn visualization (Figure 4). These 4 prototypes were evaluated in user study 1.

### Ethics Statement

The studies were approved by the institutional review boards of Wellesley College and New York University.

### User Study 1: Comparing Interactive Genetic Reports of Two People

#### Overview

Our first user study aimed to evaluate the effectiveness of each of the 4 alternative report views in comparing the genetic reports of 2 people (CrossGenomics 1.0). In particular, we investigated the following questions:


RQ1: To what extent are users able to comprehend both intraindividual (important genetic information of an individual) and interindividual information (comparing similarities and differences in the reports of 2 people)?

RQ2: How do people engage with a comparison tool for personal genomic data, and what visualization features are most helpful for comprehension?

RQ3: How does the report type impact comprehension of genomic data comparison?

RQ4: Are there any gaps between subjective and objective comprehension?

A website was developed for a between-subject experiment with the 4 alternative report views comparing the personal genomic information of 2 fictional siblings, Jamie and Alex (Figures 1-4). This fictional dataset of personal genetic reports of 2 siblings was created based on publicly available personal genomic data shared on Open Humans, using the GET-Evidence [42] interpretation. The same data were used across the different views. Using a fictional dataset to assess nonexpert *comprehension* of personal genomic data is a common practice in personal genomics studies [19,21,29].

#### Procedure

After digitally signing a consent form and responding to basic demographic questions, participants completed a tutorial on personal genomics using materials from the Personal Genetics Education Project [46]. Comprehension of pretask material was assessed by a 6-question quiz [3,22]. Those who failed to answer at least 3 questions correctly were excluded from the analysis. Users were then randomly assigned to one of the 4 experimental conditions in which they were exposed to one of the report views and interacted with it.

To assess the effectiveness of the report views, we examined comprehension of the users after their interaction with them. Participants were asked to answer comprehension questions using their report tool. The questions (see Multimedia Appendix 1) required intraindividual and interindividual information. This questionnaire was developed in consultation with a genomics expert with experience in engaging nonexperts to explore how personal genetic data relate to published research. It was adapted from an existing questionnaire [3] for use in the comparative context of this study. We then pilot-tested the questionnaire with nonexperts (Mechanical Turk workers).

In addition, participants were asked about their perception of ease of use of the tool using a 5-point Likert scale. Participants were also asked about their perceived understanding of the report. Finally, participants were asked open-response questions about how useful the visual features of the report were and what aspects should be improved. The full study 1 questionnaire is included in Multimedia Appendix 1.

#### Participants

We recruited participants via Amazon Mechanical Turk. Mechanical Turk is widely used in research aimed at a diverse nonexpert population [47,48], as well as studies of visualization perception [49]. Our goal in the experiment sample selection was not to form a representative sample of a broader public who currently undergo direct-to-consumer genetic testing (DTCGT) but rather to prepare for a relatively near future in which DTCGT is more prevalent. Prior research [50] has shown that the population of Mechanical Turk is at least as representative of the US population as other subject pools. The 99% approval rate threshold was set to ensure that participants take the response to the task before them seriously.

A total of 485 users were distributed across the following conditions: Venn view 24.5% (119/485), sunburst view 24.5% (119/485), and linear view 27.4% (133/485), and 23.5% (114/485) used a table-based view currently available to consumers (ie, control condition). Demographics of users are described in Table 1.

#### Data Analysis

We compared user responses across conditions using analysis of variance (ANOVA) and post hoc Tukey HSD tests. Responses of users to comprehension questions were scored as 1 if correct and 0 if incorrect. The sum of scores served as a comprehension measure, ranging between 0 (all responses incorrect) and 5 (all responses correct). Responses to the perceived understanding and ease-of-use questions were calculated as the mean of the responses to the respective survey items, ranging between 1 and 5. Responses to the open questions were analyzed using content analysis methods: first-level codes were developed from preliminary review by 2 independent coders. The codes were then collapsed into categories based on frequency, and themes were identified through analysis of categories. Intercode reliability based on 100% of the data was very good at 93%.

### User Study 2: Comparing Interactive Genetic Reports of Four People

#### Overview

**Figure 5:**
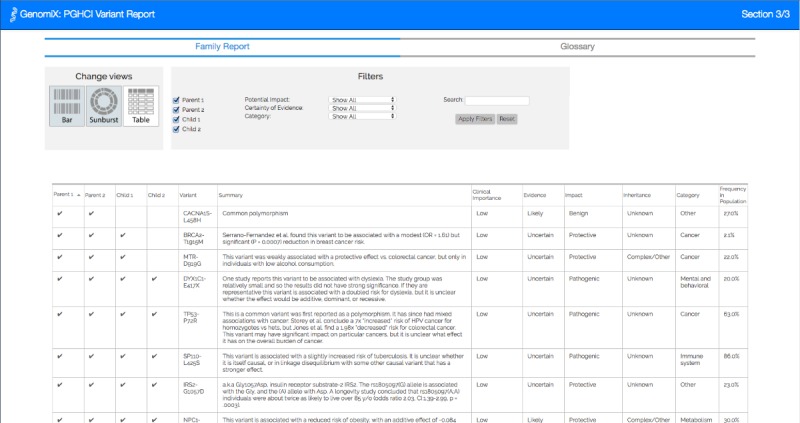
Tabular report: the table is similar to the existing GET-Evidence report, with each row representing a variant in any of the reports. Four columns were added to the table and a checkmark or a carrier indicator was used to denote the presence of a gene variant for each family member. We added a new search and filters bar.

**Figure 6:**
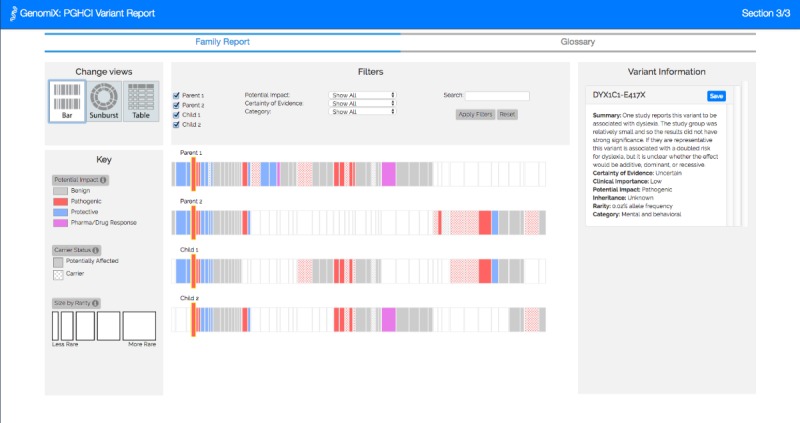
Linear visualization: each rectangle represents a gene variant and each row represents a family member’s genome. Parent 1’s variants are represented at the top, followed by Parent 2’s variants, then Child 1’s variants, then Child 2’s variants at the bottom. The colored variants in each row represent the variants of one family member.

**Figure 7:**
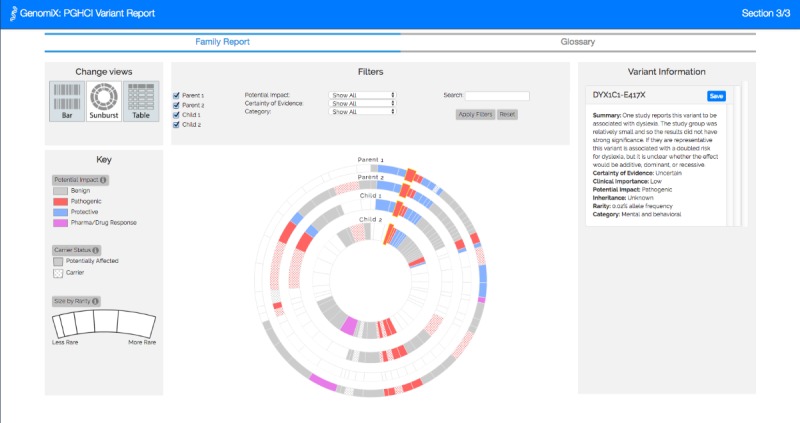
Sunburst visualization: each arc represents a gene variant, and each full circle represents a family member’s genome. The outer circle represents Parent 1’s variants, followed by Parent 2’s variants, then Child 1’s variants, and then Child 2’s variants in the inner circle. The colored variants in each circle represent the variants of one family member.

In study 2, we sought to evaluate the extension and redesign of the tool (CrossGenomics 2.0) to facilitate a comparison between 4 fictional family members, 2 siblings and their parents. We evaluated the effectiveness of each of the 3 report views (table, linear, and sunburst) in comparing the genetic reports of 4 family members, as well as a fourth view that offered users the ability to switch between the 3 other prototypes. Figures 5-7 show the interactive reports used in this study.

In particular, we expanded on our investigation of RQ1-RQ4 from user study 1 and explored an additional question:


RQ5: Does the ability to switch between different genetic data visualization, based on the information sought by the user, affect comprehension and behavior?

Similar to study 1, we assessed to what extent users were able to comprehend both intraindividual information and interindividual information. We conducted a between-subject experiment, comparing the 4 different interactive report prototypes. Each report presented the personal genomic information of 4 fictional family members, 2 parents and 2 children. The same personal genomic data, which were created based on publicly available genomic information shared on Open Humans, using the GET-Evidence [42] interpretation, were used across the different views.

#### Procedure

After digitally signing a consent form and responding to basic demographic questions, users completed the same tutorial on personal genomics and comprehension test of pretask material from study 1. Those who failed to answer at least 6 questions correctly were excluded from the analysis. Users were then randomly assigned to one of the 4 experimental conditions in which they were exposed to one of the tools and interacted with it.

To assess the effectiveness of the tools, we examined users’ comprehension using 14 comprehension questions. The questions were designed to assess users’ understanding of different concepts (impact, comparison, carrier status, category, rarity, and certainty) and required intraindividual and interindividual information. This comprehension questionnaire was developed in consultation with the same genomics expert from study 1, adapting the questionnaire from study 1.

**Table 1:** Demographic information for the 3 user studies.

Study number	Participants, N	Population	Average age (years)	Gender (female), n (%)	Compensation	Purchased DTCGT^a^, n (%)
1	485	Amazon Mechanical Turk US users with a record of at least 100 tasks at an approval rate above 99%	34.8	233 (48.0)	US $5	6 (1.2)
2	183	Amazon Mechanical Turk US users with a record of at least 100 tasks at an approval rate above 99%	35.2	99 (54.1)	US $5	9 (5.0)
3	49	Users with publicly available 23andMe data on Open Humans	51.3	17 (34)	Lottery for a FitBit Ionic Watch	49 (100)

^a^DTCGT: direct-to-consumer genetic testing.

Participants were also asked about their subjective perception of ease of use of the tool, using a 5-point Likert scale. Finally, users were asked open-response questions about the usefulness of the visual features of the report and about possible improvements. The complete study 2 questionnaire is included in Multimedia Appendix 2.

#### Participants

We recruited 183 Mechanical Turk users, who were distributed across the following conditions: linear (45/183, 24.5%), sunburst (43/183, 23.5%), and table (48/183, 26.2%), and 45 (24.5%) used a report that combines the 3 conditions with toggle functionality. All participants in the combined report switched at least once between views. Participant demographics are described in Table 1.

#### Data Analysis

Analysis was the same as in study 1, but with the addition of chi-square tests. For Q9-Q12, responses were coded as correct if the answer described a pathogenic or pharmacogenetic variant or a variant for which the family member was a carrier if the user mentioned the potential effect for offspring in their reasoning. In addition, responses to these questions were assigned a complexity score from 0 to 6 for their reasoning, with the goal of measuring ability of the users to assimilate complex information and use it appropriately in their analysis of the report.

### User Study 3: Evaluating Interactive Reports With Users’ Own Data

#### Overview

In study 3, we sought to evaluate the modification of the tool (CrossGenomics 2.1) with users interacting with their own personal genomic data. We evaluated the integration of the GenomiX single-user gene variant visualization [22] with a redesigned version of the combined view—comparing the user’s own personal genomic report to the report of one of the 4 famous people using the tabular, linear, sunburst, and Venn diagram visualizations. In doing so, we sought to draw on the trend of self-comparison with celebrities, which is increasingly evident in popular culture [4,5]. All the filter and search features from CrossGenomics 2.0 were included in this version.

In particular, we investigated the following question:


RQ6: To what extent and in what ways are users interested in comparing their genetic data with others?

#### Procedure

We created a study on Open Humans, recruiting users with publicly available 23andMe data. Participants were enrolled in our study and were assigned anonymous project member IDs. Although the study called for users with publicly available data in all communications with potential participants [51], any Open Humans user was technically able to enroll. Open Humans enables individuals to publicly share data through a multistep process (informed consent, quiz, and *opt-in* for each data type), and we discovered that some participants did not realize they had not completed all necessary steps. Thus, we created a Python script to identify which users did not have publicly available data and informed them that although they were under no obligation to make their data public, we could not generate a report unless they did. Some of these users chose to make their data publicly available, while others removed themselves from the study. We created another Python script to convert the available datasets into files with comma separated values format compatible with our visualization. We then sent messages through the Open Humans API, inviting participants to view their report and respond to our feedback form. Each user was sent a unique link that included their project member ID as a variable passed through the URL. On page load, the visualization would read the ID and load the corresponding data.

The feedback form was implemented on Google Forms. Participants were sent a link in their invitation message along with the link to the tool, and the tool itself also contained a feedback tab with the same form embedded. Users could choose whether to record their project member ID. Participants were asked about their motivation for exploring their genetics, as well as which tools they had previously used to explore their personal genetic data. In addition, participants were asked to rate the ease of use and their perceived understanding on a 5-point Likert-scale and were asked open-response questions about new insights using the tool and their interest in genome comparison. The feedback form also included a series of demographic questions. Participants could choose to provide contact details for a future study comparing their data with their real-life family dataset using our tool. The complete study 3 questionnaire is available in Multimedia Appendix 3.

#### Participants

A total of 163 reports were generated, and 137 (84.0%, 137/163) users viewed their report. Of those, 49 (30.0%, 49/163) responded to our feedback form about the tool. Users who completed the study were entered in a lottery for a FitBit Ionic Watch for completing the feedback form. We present the findings from these 49 respondents, as well as the usage data from the 38 respondents who recorded their project member ID with their feedback. All users had publicly available 23andMe data on Open Humans.

Overall, 36% of respondents (18/49) reported working in life sciences (8/49) or studying life sciences at the collegiate or higher level (16/49). Additional demographics are described in Table 1.

When asked to select their highest level of education, 8% (4/49) of respondents had a high-school diploma, 20% (10/49) of respondents had some college education, 6% (3/49) had an associate’s degree, and 10% (5/49) had a bachelor’s degree. In addition, more than half of the respondents reported having an advanced degree—29% (14/49) of respondents had a master’s degree and another 29% (14/49) had a doctoral degree. These education demographics are consistent with the description of early adopters by Rogers’ theory of the diffusion of innovations [52], which details that early adopters tend to have expert knowledge, an advanced education, and a willingness to engage in trials of new technologies.

#### Data Analysis

Responses to the 5-point Likert-scale questions for perceived understanding, ease of use, and inquiries about new insights were coded from 1 (strongly disagree) to 5 (strongly agree) for each question. Responses to the open questions were analyzed in the same methods as in studies 1 and 2. Intercode reliability based on 100% of the data was very good at 96%. Four participants submitted multiple feedback forms. In these cases, the data from the free-response questions were combined, and the most recent quantitative data from the Likert-scale responses were used.

## Results

### User Study 1: Comparing Interactive Genetic Reports of Two People

#### Comprehension and Perceived Understanding

Participants spent, on average, 19.5 min (SD 8.8) on the task of exploring the genetic reports of 2 fictional family members and responding to comprehension and perceived ease-of-use questions. The average time it took users to complete their task did not differ significantly across the 4 conditions (see Figures 1-4; *F*_3,481_=1.107, *P*=.35). However, the results of the ANOVA and post hoc corrections (see Tables 2 and 3) show differences in the effects of using the tools: objective comprehension scores of the Venn tool users and the sunburst users were significantly higher than scores of the table users (*P*<.001 and *P*=.03, respectively). At the same time, however, sunburst users reported significantly lower perceived understanding than table users (*P*=.03).

**Table 2:** Comprehension, perceived understanding, and ease of use across report types.

Report type	Comprehension	Perceived understanding	Ease of use
Venn	3.83	3.81	3.92
Sunburst	3.63	3.63	3.58
Linear	3.53	3.74	3.81
Table	3.22	3.92	3.78

**Table 3:** Significant differences in post hoc comparison.

Report types	*P* value		
	Comprehension	Perceived understanding	Ease of use
Venn and table	<.001	N/A^a^	N/A
Sunburst and table	.03	.03	N/A
Venn and sunburst	N/A	N/A	.02

^a^N/A: not applicable.

In addition, reported ease of use of sunburst users was significantly lower than that of Venn users (*P*=.02). A regression analysis revealed no significant effect of demographics and education level on comprehension, perceived understanding, and ease of use.

#### Usage

Participants used the filtering feature 12.4 times on an average. Users of the linear tool filtered significantly more than users of the other tools, filtering most by health category (8.1 times on an average). The users of the sunburst and Venn tools filtered by impact only 3.3 and 2.5 times, respectively—significantly lower than linear and table tools users (both *P*<.001).

#### Features

Qualitative data indicated that filtering was found to be the most helpful feature across all conditions, with 66.0% (320/485) directly highlighting its impact on their understanding. For example, one user noted that because of the sheer amount of information, which makes the report overwhelming at first, filtering made it “easier to at least see an overall picture of who is more predisposed to certain conditions.” Filtering also helped with specific searches, as one participant commented “...being able to filter it to just show the variants that were related to cancer helped create a clear comparison.”

Moreover, 10.3% (50/485) of the users noted that they would have liked filtering which enables to “combine filter results so that [they] can look at multiple categories at once [and could] result in a single ‘hit’.” This comment was less significantly frequIn total, 56.5% of users (51% in the combinent in the Venn relatively to the linear (*P*=.03), sunburst (*P*=.01), and table (*P*<.001).

#### Confusion

Some users found the information to be overwhelming or confusing. Specifically, the sunburst was found to be more confusing (13.4%, 16/119) than the table (5.3%, 6/114; *P*=.03). For example, one user in the sunburst condition commented that “...the filters were helpful, but...did not know where to begin and had trouble figuring out which filter to use.” In addition, 11.8% (14/119) of the Venn tool users and 10.5% (14/133) of the linear tool users found their visualization confusing; however, the differences between them and other conditions were not significant.

### User Study 2: Comparing Interactive Genetic Reports of Four People

#### Comprehension

Participants spent, on an average, 29.2 min (SD 12.5) on the task of exploring the reports and responding to comprehension and the perceived ease-of-use questions. The average time it took users to complete their task did not differ significantly across the 4 conditions (see Figures 5-7; *F*_3,177_=0.672, *P*=.57). However, the results of the ANOVA (see Tables 4 and 5) indicate a significant effect of report type on objective comprehension for the 3 conditions (*F*_3,176_=5.538, *P*=.001). Post hoc comparisons using Tukey HSD test indicated that the mean score for the table and combined report type were significantly higher than the mean score for the linear report.

Further analysis of scores by individual questions indicates a significant effect of report type on the scores of only 3 of the 14 comprehension questions (Multimedia Appendix 2). For RQ6, “Which child shares the most cancer-related variants with Parent 1?,” participants in the combined condition (mean 0.89, SD 0.32) scored significantly higher than those in the linear condition (mean 0.77, SD 0.48, *P*=.04; *F*_3,177_=2.810, *P*=.04). For RQ7, “Parent 1 has _____ cancer-related variants than Parent 2,” participants in the linear condition (mean 0.44, SD 0.50) scored significantly lower than those in the combined (mean 0.71, SD 0.46, *P*=.04) and table conditions (mean 0.79, SD 0.41, *P*=.01; *F*_3,177_=4.754, *P*=.01). For RQ8, “Which variants are not expected to affect Child 1 themselves, but may affect Child 1’s future children?,” participants in the combined condition (mean 0.67, SD 0.48) scored significantly higher than those in the linear (mean 0.33, SD 0.48, *P*=.01) and sunburst conditions (mean 0.40, SD 0.50, *P*=.04), and participants in the table condition (mean 0.65, SD 0.48) scored higher than those in the linear condition (mean 0.33, SD 0.48, *P*=.01; *F*_3,177_=2.810, *P*=.04).

**Table 4:** Mean scores and SD for comprehension, perceived understanding, and ease of use for participants in each of the 4 conditions.

Report type	Comprehension, mean (SD)	Perceived understanding, mean (SD)	Ease of use, mean (SD)
Linear	9.55 (2.94)	3.69 (0.86)	3.93 (0.91)
Sunburst	10.58 (3.11)	3.69 (0.69)	4.07 (0.64)
Table	11.17 (2.25)	3.78 (0.84)	4.09 (0.80)
Combined	11.67 (1.92)	3.86 (0.67)	4.32 (0.57)

**Table 5:** Post hoc differences from the linear tool, with significant and trending *P* values.

Report types	*P* value		
	Comprehension	Perceived understanding	Ease of use
Linear and combined	.001	N/A^a^	.07
Linear and table	.01	N/A	N/A

^a^N/A: not applicable.

Overall, we find that the use of the combined report, which enables users to switch between report types based on their information needs, is associated with significantly higher comprehension level. We also find that using the table-based report leads to better comprehension than each of the noncombined visual reports.

#### Subjective Experience

Despite the difference in comprehension scores across conditions, the results of the ANOVA suggest no significant effect of report type on the perceived understanding (*F*_3,177_=0.502, *P*=.68) or ease-of-use scores (*F*_3,177_=2.083, *P*=.10; see Table 5), with the exception of a trending difference between the combined and linear conditions, indicated by post hoc analysis (*P*=.07). There was a strong correlation between perceived understanding and ease-of-use scores (*r*=.735, *P*<.001), a moderate correlation between ease of use and comprehension (*r*=.328, *P*<.001), and a weak correlation between perceived understanding and comprehension (*r*=.170, *P*=.02).

#### Complexity

Correct responses to Q9 to Q12 were assigned a complexity score from 0 to 6 for their reasoning, with points assigned when users reference each of the following concepts: potential impact, the certainty of evidence, carrier status, rarity, and clinical importance, with a final possible point for synthesis of information. In 3 questions (Q10-Q12), which inquire about which variant each family member would be most likely to discuss with their health care provider, there was a significant effect of report type on the complexity score of the correct responses (Tables 6-8). Post hoc comparisons indicated that users of the combined report had significantly higher complexity scores for these questions than the users of the linear tool. Further analysis by individual concepts revealed that the users of the combined report mentioned clinical importance significantly more than users of the linear tool in their correct responses for all 4 questions. Users of the combined report also mentioned certainty of evidence significantly more than the users of the linear tool for Q10 and Q11.

#### Demographics

A regression analysis revealed no significant effect of demographics on comprehension or subjective experience.

#### Usage

**Figure 8:**
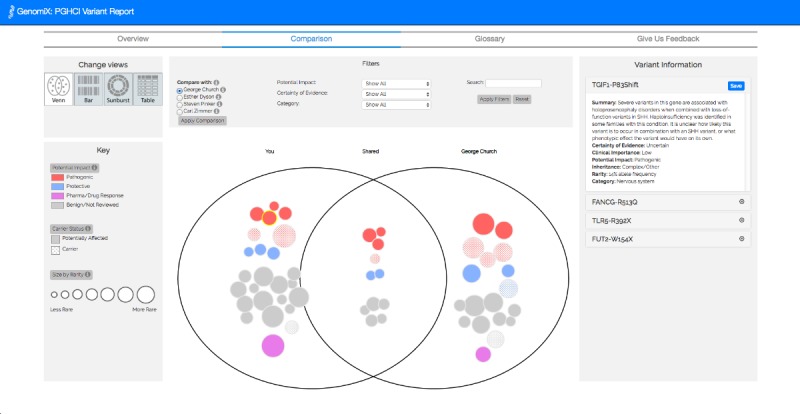
Comparison tab of CrossGenomics 2.1, where users can toggle between the four comparison visualizations using the buttons under “Change Views”.

Participants in the combined report switched on an average 5.16 times between views (SD 3.4, minimum=1, maximum=15). Moreover, 40% (18/45) of users switched views more than 5 times (see Figure 8). Out of the 45 users who interacted with the combined tool, 34 users primarily used the table, seven users primarily used the linear tool, and 4 users primarily used the sunburst tool. Furthermore, 17 of the 45 users (38%) spent more than 90% of their time exploring and answering comprehension questions using one of the 3 views. Within the combined condition, no correlation was found between the amount of switching between views and either comprehension, perceived understanding, or ease of use.

**Table 6:** Average combined complexity scores in correct responses to question (Q)9 to Q12 by condition.

Report type	Q9		Q10		Q11		Q12	
	Score	*P* value	Score	*P* value	Score	*P* value	Score	*P* value
Linear	1.40	.55	1.20	.51^a^	1.21	.58^a^	1.25	.59^a^
Sunburst	1.47	.72	1.63	.92	1.69	.77	1.43	.78
Table	1.79	1.10	1.75	1.16	1.69	1.22^a^	1.70	1.15
Combined	1.70	.74	2.05	.81^a^	1.88	.88	1.89	.84^a^

^a^Significant difference between the linear and combined conditions.

**Table 7:** Analysis of variance results to question (Q)9 to Q12.

Question	*F* (3,177)
Q9	1.918
Q10	6.620
Q11	3.948
Q12	4.368

**Table 8:** Post hoc differences between linear and combined reports for question (Q)9 to Q12.

Report types	*P* value			
	Q9	Q10	Q11	Q12
Linear and combined	.13	<.001	.01	.01

While exploring the report, across the 4 conditions users applied on average 7.60 filters (SD 10.8, minimum=0, maximum=111) and searched the report 5.5 times (SD 5.8, minimum=0, maximum=28). There was no significant effect of condition on the amount of filtering (*F*_3,177_=1.599, *P*=.19) or searching (*F*_3,177_=0.474, *P*=.70). No correlation was found between the number of filters applied and comprehension, perceived understanding, or ease of use. There was a weak correlation between the number of searches and both perceived understanding (*r*=.204, *P*=.01) and ease of use (*r*=.171, *P*=.02).

#### Report Features

Users were also asked to describe, in open-ended questions, which features were most helpful for understanding and comparing the reports, as well as how the tool can be improved.

Overall, users across all conditions liked the filter and search functionalities and suggested simpler language and more filtering options as improvements. In total, 56.5% (104/183) of users (23/45, 51% in the combined condition, 24/45, 53% in the linear condition, 21/43, 49% in the sunburst condition, and 35/48, 73% in the table condition), χ^2^_3_ (N=181)=7.009, *P*=.07, reported filtering to be one of the most helpful features. In the words of one user in the combined condition, saying, “The ability to filter by individual and category of gene (pathogenic and protective) made navigating the data much easier.”

Furthermore, 33.25% (61/183) of users (13/45, 29% in the linear condition, 12/43, 28% in the sunburst condition, 14/48, 29% in the table condition, and 21/45, 47% in the combined condition), χ^2^_3_ (N=181)=4.956, *P*=.18, also reported that the search functionality was helpful for interpreting the reports. As one user in the combined condition wrote:

Being able to search and filter out the various subjects was the most helpful to me. Being able to only focus on the information that I was interested in made the process much easier.

Furthermore, 22% (10/45) of users in the combined condition explicitly stated they liked being able to switch between representations. A user emphasized this by saying, “I also enjoyed being able to switch from table view to bar view to easily understand the information better.”

A different user noted the different usage for each view, stating:

The bar graph is easiest when lining up which family members have which genomes, and the sunburst graph was easiest when just filtering in/out things in general. The table is nice, but visually not as pleasing, but could be potentially the most useful especially if you don't have the ability to have an interactive graph.

Another user shared:

The different visualizations made it easier to understand the data in some situations [for example, the bar chart made it easier to understand quickly which family members shared what variants, whereas the table made it easier to learn about each variant].

Overall, 19.4% of all users (9/45, 20% in the combined condition, 11/45, 24.4% in the linear condition, 7/43, 16.3% in the sunburst condition, and 8/48, 16.7% in the table condition) reported difficulty with medical terms used in the report. One user, who viewed the tabular report, suggested, “Some of the explanations could be written in a more accessible manner.”

In addition, 10.4% of users (5/45, 11.1% in the combined condition, 2/45, 4.4% in the linear condition, 5/43, 11.6% in the sunburst condition, and 7/48, 14.6% in the table condition) reported requirement for more filtering options. The differences in proportion between the conditions were not significant.

### User Study 3: Evaluating Interactive Reports With Users’ Own Data

#### Previous Use of Genome Tools

All 49 users had their genome mapped by 23andMe. Unlike the users of our previous 2 studies, we classified these 49 users as expert users, as they had previous experience of exploring their genome. Furthermore, 88% (43/49) of those users had also used at least one of the following tools to explore their results: Promethease (41/49), SNPedia (30/49), Google (17/49), ClinVar (17/49), GET-Evidence (15/49), PubMed (15/49), Wikipedia (14/49), Genevieve (14/49), and OMIM (10/49).

#### Motivation

We asked participants to rank the personal importance of 8 potential reasons for exploring information about their genetics on a 5-point Likert scale from *Not at all* to *Extremely* (Table 9).

#### Usage

A total of 78% (38/49) participants provided their project member IDs, which allowed us to identify their specific usage data from the tracking logs on our tool. The usage data of these users are presented here.

##### Visualizations

All 38 users began on the comparison tab, with their genomic report compared with the report of George Church by default. Of these, 68% (26/38) viewed the overview report and 63% (24/38) viewed the glossary. Within the comparison tab, 79% (30/38) users viewed all 4 visualizations, 8% (3/38) users viewed only 3, 5% (2/38) users viewed only 2, and 8% (3/38) users did not switch visualizations at all. As the default visualization on page load, all users viewed the Venn diagram visualization. In addition, 89% (34/38) users viewed the sunburst visualization, 87% (33/38) users viewed the linear visualization, and 84% (32/38) users viewed the tabular report. Moreover, 58% (22/38) of the users compared their data with all 4 comparison genomes, whereas 16% (6/38) users did not change comparison genomes.

##### Variants

Users clicked on a total of 1609 variants in the interactive sunburst, linear, and Venn diagram visualizations. In addition, 124 variants were saved by 13 users. The majority of variants were clicked and saved in the Venn diagram, the default view of the tool on page load.

##### Filtering

The comparison and single-user visualizations also included a filter bar, which affords users the ability to filter variants—by certainty of evidence, potential impact, and category—and search for a specific term. In the comparison view, 24 users applied 209 filters and searches. Of these, 30 certainty of evidence filters were applied, 29 of which were for *well-established* variants. A total of 44.4% (93/209) filters included a filter for potential impact, 20.0% (42/209) for pathogenic variants, and 17.2% (36/209) for variants affecting drug response of a user. Another 14.3% (30/209) filters specified a certain health category, such as *Cancer* or *Metabolism*. Four users also searched for specific terms, such as *Alzheimer*, in their data.

#### Perceived Understanding

In this study, 12% (6/49) of respondents reported they would need the help of a health care professional to better understand their results (on a 5-point Likert scale, mean 2.36, SD 1.03). In addition, 41% (20/49) reported feeling that the report gives them a firm grasp of their health and genetics (mean 2.98, SD 1.15,). On the goal of communicating uncertainty, 82% (40/49) felt they could grasp the extent to which the knowledge regarding different variants is certain or uncertain (mean 3.92, SD 0.88).

#### Ease of Use

In this study, 84% (41/49) of respondents reported that they found the information in the report to be presented in a clear and accessible manner (on a 5-point Likert scale, mean 3.94, SD 1.04). In addition, 65% (32/49) found the glossary helpful (mean 3.60, SD 1.05), and 39% (19/49) found the ability to save variants helpful while interacting with the report (mean 3.22, SD 1.13).

#### Report Features

When asked which features were most helpful for understanding the report, users mentioned the usefulness of filtering in both the comparison and single-user visualizations. One user wrote:

I think most reports fail to grab attention except for a few variants that are highlighted. Sorting by certainty helped that some here.

**Table 9:** Average personal importance for potential reasons for exploring information about their genetics, 5-point Likert scale (not at all, slightly, moderately, very, and extremely).

Reason	Mean (SD)
To learn personal disease risk or health-related information	3.86 (1.14)
Curiosity	4.40 (0.81)
To contribute to research	4.28 (0.81)
Interested in distant ancestry (race or ethnicity)	3.14 (1.50)
Learning more about my family origin and recent ancestry	3.20 (1.46)
To provide disease risk information for children and other family members	2.78 (1.40)
To learn more about myself	4.22 (1.04)
Understanding these data for professional purposes	2.00 (1.20)

Users also commented on the value of toggling between different views. In the words of one user:

I specifically liked the ability to see the data presented in all three visualizations. The information for each particular variant was succinct and easy to follow.

Several users also commented that although they enjoyed the visualizations, the tabular view offered a valuable overview to all the descriptions at once. One user wrote:

I liked the table view the best, I could just read straight down, without having to move around page...The visual was interesting but I spent more time on table view.

When asked how they felt the report could be improved, several users mentioned that they wished they could print their report, while others suggested a more robust tutorial. In the words of one user, “[I] would like to be able to download the variant report in pdf format to refer to offline.” Several users also mentioned that they wished the tool began on their report, instead of the comparison tool.

#### New Insights

In this study, 49% (24/49) of the users reported learning new insights and information about their genetics using this tool that they had not noticed in previous reports (on a 5-point Likert scale, mean 3.16, SD 1.31). In addition, 43% (21/49) also reported that the visualization changed their understanding of their report (on a 5-point Likert scale, mean 3.00, SD 1.21). When asked to elaborate on what new insights and information about their genetics they learned from this visual report, some users reported discovering new variants that they had not noticed in previous reports. For example, one user wrote, “FUT2-W154X, ADA-D8N, and DPYD-M166V were all variants I hadn’t noticed before and found interesting.” Other users commented on the understandability of this report as compared with others, stating, “The way other reports or worded/written, I have hard time understanding if I have or don’t have [a variant], very confusing.” Other users reported that they did not learn anything new, as they had previously poured over their report, but still found the experience worthwhile. In the words of one user, “I had seen most of this information in previous reports, but I did enjoy the interactive visualization.”

#### Comparison

A total of 35% (17/49) reported learning something new from comparing their data with another person (on a 5-point Likert scale, mean 2.63, SD 1.30). When asked to elaborate on what they learned, 24% (12/49) expressed amazement at the number of shared variants between themselves and the comparison genomes. Others reported that they viewed the comparisons out of curiosity but did not learn anything new. In the words of one respondent, “I looked at the comparisons but they did not engage me because I do not know the people.”

When asked if there are any other potential people with whom they would like to compare their data, 51% (25/49) responded with at least one family member. In the words of one participant:

I liked the Venn diagram and I would use it to compare with other family members. This would be awesome if I could load even more data—for example, for our family of four—to see what we passed on to our kids.

In fact, more than half of the users (51%, 25/49) recorded their project member ID or email address to be contacted with opportunities to compare their data with that of their family members using our tool. In addition, 10% (5/49) participants lamented that they would have liked to compare themselves with specific members of their family who have passed away or are not interested in genetic testing. When asked what questions they would like to explore in comparison with these individuals, 24% (12/49) mentioned an interest in inheritance patterns between family members. In addition, 10% (5/49) of users mentioned an interest in exploring potential health risks through family comparison.

## Discussion

### User Study 1: Comparing Interactive Genetic Reports of Two People

We found that all views allowed users to complete the task of comparing 2 personal genomic reports with at least moderate domain comprehension of both intraindividual and interindividual information (see Table 2; RQ1). The sunburst- and Venn-based prototypes led to significantly higher domain comprehension, compared with the linear prototype and the commonly used tabular report (RQ3). However, we identified gaps between objective and subjective comprehension, as sunburst users reported significantly lower perceived understanding and higher levels of confusion (RQ4).

Although the Venn tool appears to be most effective, it is limited in scalability, as it can only show the data of 2 users at a time. The other prototypes, in particular the sunburst and the linear tools, can present data of a larger number of users. In study 2, we aimed to further explore the utility of these prototypes in facilitating domain comprehension when comparing a larger number of genomic reports. Our redesign includes genomic reports from 4 biological family members. As expanding the number of compared reports to 2 was a planned feature, we decided to first evaluate and learn from the comparison of 2 personal genomic reports.

Through analysis of the open-ended responses, we learned that users viewed the ability to filter and search the data as most helpful (RQ2). We found a relatively consistent pattern of usage of these features across the 4 report types. These findings led us to implement a search tool in our next design iteration across all different visual reports and to redesign our filtering functionality to allow users to combine filters. In study 2, we investigated the role of search and filtering when there is an increase in the information displayed.

#### Redesign

There were 3 redesign iteration stages leading to CrossGenomics 2.0. The first iteration stage focused on expanding the visualization by presenting the genetic reports of 2 family members to 4. This resulted in the linear visualization having 4 linear bars and the sunburst visualization having 4 concentric rings. We did not include the Venn visualization in this redesign.

Due to increase in the information displayed, we added a filter to show or hide certain family members so that users could focus on certain individuals without being distracted by others. We also added tutorial tooltips that briefly describe the function of the key, filters, and variant information panels. On the basis of feedback gathered from the first study, we increased the size of the panel where variant information appeared so that scrolling was not required. We also modified the filtering panel so that users could combine filters and see all of the filters they had selected.

We tested this version in a pilot study with 25 nonexpert users (Mechanical Turk workers) per condition. We found that users in the table condition used the browser search function to find particular important keywords (eg, cancer) and found the search to be helpful. In addition, we identified that users were not saving variants for revisiting the information.

Informed by these findings and by the findings from study 1, we implemented a search tool that was consistent across the different visual reports and allowed searching for keywords within a visual report and highlighted the *save* button by changing the button color from gray to blue. We also introduced a new visual report that allowed users to switch between the 2 visual reports (linear and sunburst) and the table report. This version was subsequently tested with 10 Mechanical Turk users per visual report.

Results further highlighted the important role of the search feature, as well as a need to combine the application of the potential impact and certainty of evidence filters with the category filter. Results also indicated that users still rarely *saved* variants. As such, we moved both the search and the category filters to be part of the core filter functionality, allowing a user to combine all of them together. We also added a tooltip prompting users to *save* a variant on the first selection.

Figures 5-7 show the 3 redesigned reports, integrated into an interactive tool, which allows users to switch between these 3 views. These 3 prototypes were evaluated separately in comparison with the combined tool in study 2.

### User Study 2: Comparing Interactive Genetic Reports of Four People

Users across all conditions completed the task of comparing 4 personal genomic reports, demonstrating at least moderate domain comprehension of both intraindividual and interindividual information (see Table 4; RQ1). A key finding from this study is that users in the combined condition did, in fact, switch between the views presented and demonstrated higher comprehension levels, as well as more complex reasoning (RQ5). More than 20% of the users in this condition explicitly mentioned switching between views as a helpful feature (RQ2).

We also found that using the table-based report led to better comprehension than the noncombined visual reports (RQ3). In particular, the linear visualization performed the worst compared with the combined tool and with the table-based report. The sunburst tool did not perform significantly better or worse compared with any of the tools. In addition, we found that although all users of the combined tool switched at least once between visualizations, about three-fourths of these users chose to primarily use the table-based report to answer comprehension questions. A possible explanation for these results may be the lack of familiarity and experience of nonexperts with visual tools, in contrast with familiarity with table-based reports.

Despite the difference in comprehension scores across conditions, the results suggest no significant effect of report type on the perceived understanding. This indicates a gap between objective and subjective comprehension (RQ4).

On the basis of these findings, which indicate that overall the combined condition of CrossGenomics 2.0 is effective for comparing personal genomes across individuals, we adapted this tool to study (in study 3) how Open Humans participants use it to explore their own personal genomic data.

#### Adapting CrossGenomics for User Data

Following user study 2, the combined visual report from CrossGenomics 2.0 was modified to load real 23andMe data that were shared *publicly* on Open Humans profile of a user. This allowed us to engage a pre-existing community where individuals are publicly sharing genetic data, including nearly 1000 individuals who already publicly share these data in the Harvard Personal Genome Project [42] or openSNP projects [53]. This new prototype of CrossGenomics allowed users to compare their own report with one of the 4 publicly shared genomes of famous scientists and thought leaders on genetics (George Church, Esther Dyson, Steven Pinker, and Carl Zimmer). The tool provided users with 4 different views: tabular, linear, sunburst, or Venn diagram visualizations.

**Figure 9:**
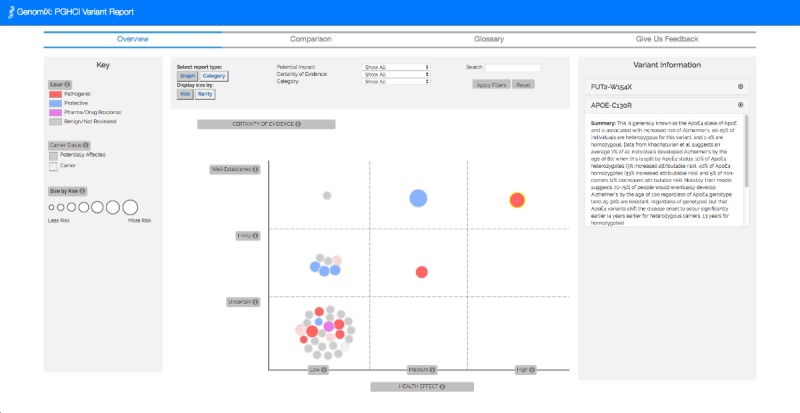
Overview tab of CrossGenomics 2.1 in graph view, where users can view their own personal genomic data graphed by certainty of evidence and potential health effect in our single-user GenomiX visualization.

**Figure 10:**
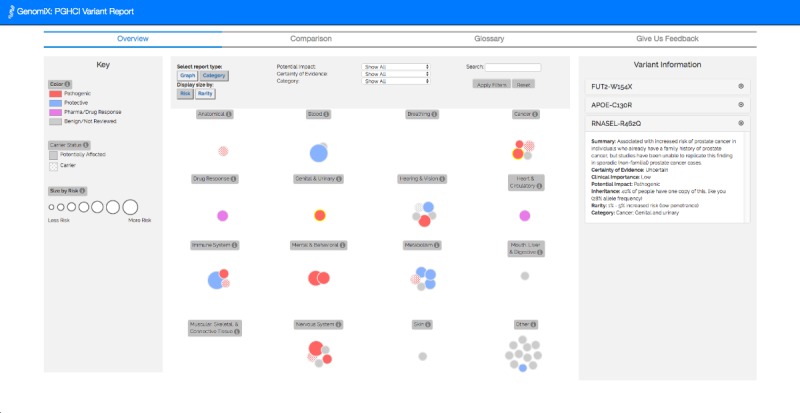
Overview tab of CrossGenomics 2.1 in category view, where users can view their own personal genomic data by health category in our single-user GenomiX visualization.

The genome to which the data of a user are compared could be selected using a radio button. This modified comparison tool was integrated with the single user GenomiX [22] gene variant report, presented in a separate tab, to create CrossGenomics 2.1—a tool that allows users of Open Humans to explore their own 23andMe data and compare it with 4 other genomes. Figures 8-10 show the GenomiX tool integration and the 4 visualizations in the comparison tool.

### User Study 3: Evaluating Interactive Reports with Users’ Own Data

Results indicate that the majority of participants found the report to be presented in a clear, accessible, and comprehensible manner. Findings also indicate that the ability to switch between views is particularly helpful. In addition, participants highlighted filtering, sorting, and saving variants as important features. These findings shed light on how people engage with a comparison tool for personal genomic data and what features are most helpful for comprehension. More than half of participants expressed interest in comparing their personal genomic data to that of a family member using our comparison tool, expressing motivation to explore inheritance patterns and health risks. Fewer users were interested in the comparison of their data to the “famous genomes” provided. Over one third of the participants reported learning something new from comparing their data to another, mostly highlighted the similarities between their own report and the others’ reports.

These findings indicate an interest in comparison and highlight the ways in which nonexperts seek to compare their genetic data to others’ (RQ6). Findings also suggest that users are able to comprehend both intra-individual (important genetic information of an individual) and inter-individual information (comparing similarities and differences in the reports of two people; RQ1).

### Principal Findings

Taken together, the 3 studies advance the prior personal informatics research by offering a novel approach to enabling user engagement in the transition between integration, reflection, and action [10-12]. Overall, we contribute to research on personal and health informatics in 2 central ways: first, we advance the literature in the specific domain of personal genomics by offering and evaluating a novel design for interpersonal, comparative, and genomic information reporting tool. Second, the findings underscore the need to understand and design for the social aspect of deeply personal information and call for careful theoretical consideration of design decisions in this space.

The first prototype (study 1) served as a proof-of-concept for eliciting users’ feedback. As a result of the reported findings, we redesigned the tool by introducing a consistent search tool across the visual reports and by adding a new feature to enable users to switch between the reports. Study 2 was an extension of study 1, enabling users to compare up with 4 people, which further corroborated the usefulness of the redesign done after study 1. Building on both studies and users’ feedback, in study 3 we sought to enable Open Humans participants to explore their own personal data. The findings offer a new perspective on how users can integrate and reflect on data, both in a personal and social context: (1) highlighting features and visualizations that show strengths in facilitating user comprehension of genomic data, (2) demonstrating the value of affording users the flexibility to examine the same report using multiple perspectives, and (3) emphasizing users’ needs in comparison of genomic data. In the following paragraphs, we discuss the findings in detail, as well as the ethical considerations and implications for the design of tools for multiuser engagement with complex multidimensional data.

In user study 1, we evaluated 4 alternative views for CrossGenomics, a novel interactive tool enabling nonexpert users to explore what gene variants they share with others and what sets them apart. We found that the sunburst- and Venn-based prototypes led to significantly higher domain comprehension, compared with the linear prototype and the commonly used tabular report (RQ1). Although the Venn tool appears to be particularly effective, it is limited in scalability, as it can only show the data of 2 users at a time. The other prototypes, in particular the sunburst and the linear tools, can present data of a larger number of users. A trade-off therefore exists between the utility of the tool and its user coverage per comparison session.

We also found that users viewed the ability to filter and search the data as most helpful for comprehension and exploration (RQ2). We found a relatively consistent pattern of usage of these features across the 4 report types, suggesting these features are important for both visualization and table-based reports. These findings highlight the benefit of providing features that allow users to focus on and switch between relevant subsets or dimensions of the information. Recent study by Feng et al has corroborated this finding, suggesting that the presence of text-based search influences information-seeking goals of people and can alter both the data explored and the ways in which users engage with it [54]. In addition, they found the effects of the search are amplified in visualizations where the users are familiar with the underlying dataset, such as the expert users in our third study.

In user study 2, we also found that using the table-based report led to better comprehension than the noncombined visual reports (RQ3). In particular, the linear visualization performed the worst compared with the combined tool and with the table-based report. This stands in contrast to the popularity of the linear alignment visualization in tools for experts [27,29-31,45]. In addition, we found that although all users of the combined tool switched at least once between visualizations, about three-fourths of these users chose to primarily use the table-based report to answer comprehension questions. A possible explanation may be the lack of familiarity and experience of nonexperts with visual tools, in contrast with familiarity with table-based reports. This highlights a need to guide users carefully through the use of visual reports. Recent research has proposed and evaluated new ways of providing such guidance [55].

The findings from user study 1 also suggest a discrepancy between objective and subjective knowledge of users—what users know and how much they think they know [56-58] (RQ4). We found that the sunburst tool that was associated with higher domain comprehension (objective knowledge) compared with the tabular report was also associated with significantly lower perceived understanding (subjective knowledge), which may be explained by the users’ comments about the sunburst tool as being significantly more confusing or overwhelming. Similar discrepancies were reported in other domains in which information tools were used for clarifying complex information [59]. For example, Gunaratne et al [60] found that exposure to social annotations of financial disclosure documents increased performance but reduced perceived understanding. A possible explanation for such discrepancies is that the more knowledgeable users become, the more they realize how complex the information presented to them is and realize how much of it they do not understand. Although we did observe a similar trend in the second study, the discrepancy between objective and subjective knowledge was not statistically significant. Further research is needed to explore these differences.

A key finding from user study 2 is that users who were given the flexibility to choose how personal genomic data are presented to them (through the ability to switch views) did, in fact, switch between the views presented and that these users demonstrated higher comprehension levels and were able to offer more complex reasoning to justify their choices based on evidence from the visualizations (RQ5). Thus, the findings suggest that providing users’ autonomy to pursue their information needs based on their preferences, as well as to explore data from multiple perspectives lead to better comprehension and perceived ease of use.

The findings from user study 3 suggest that a majority of our users have an interest in comparing their personal genomic data with that of a family member using our comparison, and fewer users were also interested in the comparison of their data with the *famous genomes* provided, beyond a one-time exploration (RQ6). Of the participants who reported gaining new insight from the comparison tool, most highlighted the similarities between their own report and the report of the other person. Building on the concept of biosociality—how people form social ties around health conditions caused or influenced by genetic characteristics—one future direction to explore would be to focus on people with known similar conditions or similar communities for comparison.

The studies and their findings also raise ethical issues. One such concern stems from unique combination of unchanged data and changed interpretation of personal genomic information, driven by the new findings resulting from advances in genetic research. By making individual and social personal genomic data available, comparable, and interpretable, users can engage continuously with unchanging *data for life* [61]. However, access to such data in the context of personal information can inform everyday choices of users [61],and access to comparative information about others may lead to information surveillance [1], which is particularly concerning in situations where future genetic research may suggest previously unknown relationships between genetic data and health conditions. As a result, new implications about users’ data could be shared with others without the original user’s control. Moreover, the promise of empowerment, often pervasive in discussions of health self-tracking and personal informatics [62], and the critical perspectives approach to this promise [63,64,65] are all the more evident in the case of personal genomics, where individuals’ agency is often limited and where powerful institutional actors (including state agencies and financial institutions) have the potential to gain from access to information and its present and future interpretations.

### Design Implications

On the basis of our findings from the 3 studies outlined above, we propose a number of design implications, some of which are specific to the personal genomics context, and some more general, concerning insights for multiuser engagement with complex multidimensional data.

The context of personal genomics in which data of individual family members is directly relevant to other family members, calls for both holistic and focused points of view. In that sense, the trade-off between scalability and effectiveness evident in the performance differences between the Venn and the sunburst visualizations reflects a design consideration for practitioners; some design cases may call for prioritization of better understanding—both objective and perceived—such as in the case of rich comparisons between siblings where a Venn approach would be most effective, whereas others may require a broader but less rich perspective in which a sunburst approach might work better.

Beyond personal genomics, insights from this study can inform other approaches to visualizing personal data. For example, the approaches presented here to visualizing genomic information, illustrate a method to engage with Lived Data [66], allowing users to explore their data in a broad and socially relevant context. They also offer a perspective on Lived Data that transition from temporal changes of data to temporal changes of potential interpretations. These approaches can be used to compare cuts of interest (collected data of some shared feature) across users [13] and serve as a practical way to represent the interconnected self within the context of relevant others [67].

The design approach presented in studies 2 and 3, which integrates multiple views, underscores the need for autonomy for users to pursue their information needs and to explore data from multiple perspectives. Such an approach leads to better comprehension and perceived ease of use. To that end, 2 types of features are needed that will help users to further reap the benefits from multiuser personal genomic comparison tools. These include tutorials to carefully guide users through the use of visual reports and features that allow users to focus on and switch between relevant dimensions of the information, particularly with users who are familiar with the underlying dataset [54].

Two key issues concern possible adverse implications for the proposed approach. First, ability of nonexperts to engage with their personal information in a social context carries a concern of *misinterpretation and information overload*, which may dilute the understanding of the data and undermine the original goals of the tools designed. More research on filtering mechanisms, such as the approach proposed by Jones and Kelly [68], is needed to ensure effective engagement with socially contextualized personal data. A second issue concerns *privacy* —the effectiveness of multiuser personal genomic comparison tools arises from the juxtaposition of multiple genetic profiles, yet that same aspect of their value to users implicitly assumes that users would be interested in and willing to share their data with other family members. This assumption may hold for many families but not for all. Making personal genome comparison tools available to users may lead to undue expectations, or even pressure, among family members concerning data sharing. This concern also highlights a need to develop new mechanisms for users to understand the risks associated with publicly sharing genetic data, as well as to control what aspects of their data to share, with whom, and for what purposes.

### Limitations

Although the design and evaluation of CrossGenomics offer insights into the design of future personal genomics exploration and comparison tools, there are limitations to this study that should be considered in future research. First, we did not assess the fluency and familiarity of participants with visualization techniques, so we cannot speak about the effect of this experience on comprehension, subjective experience, and behavior in interacting with these 4 report types. Second, the analytical validity and clinical utility of DTCGT have been debated [69]; however, CrossGenomics is a tool for exploring and facilitating health information-seeking behavior [70], rather than a clinical tool, and as such is not attempting to provide or assess clinical utility. Third, a majority of the users of the combined report primarily used the table, which could be because of the nature of our sample. Namely, the users in our first and second study most likely exhibited less exploratory behavior than those of the potential nonexpert users interacting with their own personal genetic and family data. Our third study, which included users interacting with their own data, did not offer the opportunity for a meaningful comparison between family members. In addition, our sample of early adopters is not representative of the general public, as it is limited to individuals with genetic data who also chose to share it publicly. Although it is rare to find families with public genetic data, our future study will engage nonexpert users comparing their own personal genetic data, with the data of their family members.

### Conclusions

The rapid increase in the availability of complex personal genomic data to nonexpert users poses research and practice challenges and opportunities. The interpretation of such data may impact lifestyle decisions, emotional state, and well-being of users and their family members. However, research on interaction of users with personal genomic data is still limited. The familial nature of personal genomic data highlights the need for tools to enable nonexperts to explore not only their own data but also to compare and contrast their data with data of other biological family members, who share common genetic characteristics.

Beyond the contribution of this research to the personal genomics domain, our study makes the following contributions: (1) presenting the design and evaluation of tools that facilitate multidimensional, multiperson comparisons, (2) analyzing the differences between comprehension and perceived understanding, leading to a better understanding of discrepancies between subjective and objective knowledge, and (3) highlighting design considerations for multiuser engagement with complex multidimensional personal data.

Empowering nonexpert users by facilitating a better understanding of their genetic characteristics and that of their families is an important step in helping people be more self-informed. We intend to further evaluate CrossGenomics with nonexpert users comparing their own personal genetic data with the data of their family members. Ultimately, our goal is to make CrossGenomics a free tool available for the Open Humans community. Personal genomics is a domain in which interactive technologies can make a real difference in the lives of users, and the studies reported here advance both research and practice in this direction.

## Appendix

Multimedia Appendix 1 User Study 1 Questionnaire.

Multimedia Appendix 2 User Study 2 Questionnaire.

Multimedia Appendix 3 User Study 3 Questionnaire.

## Abbreviations

ANOVA: analysis of variance
API: application programming interface
DTCGT: direct-to-consumer genetic testing
HCI: human-computer interaction
