# Supplementary material for: Exploring Genetic Data Across Individuals: Design and Evaluation of a Novel Comparative Report Tool
Source: J Med Internet Res. 2018 Sep 24;20(9):e10297. doi: 10.2196/10297 (PMC6231826; doi:10.2196/10297)
Supplement: Multimedia Appendix 1 [file jmir_v20i9e10297_app1.pdf]

Appendix 1: User Study 1 Questionnaire

1. How old are you?
2. What is your gender?
3. Level of education completed:
4. Do you work in the life sciences?
5. Did you study life sciences at a collegiate or higher level?
6. Have you had your genome mapped before?
7. Which genetic testing service did you use?
8. What reports or tools did you use to view and learn from your personal genomic data?
9. How many pathogenic variants are shared by Jamie and Alex?
10. How many protective variants are shared by Jamie and Alex?
11. Based on the information above, Jamie has _______ pathogenic variants compared to Alex.
12. Based on the information above, Jamie has _______ protective variants compared to Alex.
13. Based on the information above, Jamie is a "carrier" for _______ variants compared to Alex.
14. Which variant would Jamie be most likely to discuss with a healthcare provider, and why?
15. Which variant would Alex be most likely to discuss with a healthcare provider, and why?
16. Based on the information above, Jamie has _______ variants reported to affecting cancer risk compared to Alex.
17. Which of the following do Jamie and Alex BOTH have? (Increased Alzheimer's risk)
18. Which of the following do Jamie and Alex BOTH have? (Increased risk of esophageal cancer)

19. Which of the following do Jamie and Alex BOTH have? (Reduced risk of age-related macular degeneration)
20. Which of the following do Jamie and Alex BOTH have? (Reduced risk of obesity)
21. Which of the following do Jamie and Alex BOTH have? (Stomach flu resistance)
22. Which of the following does Jamie have – but NOT Alex? (Increased Alzheimer's risk)
23. Which of the following does Jamie have – but NOT Alex? (Increased risk of esophageal cancer)
24. Which of the following does Jamie have – but NOT Alex? (Reduced risk of age-related macular degeneration)
25. Which of the following does Jamie have – but NOT Alex? (Reduced risk of obesity)
26. Which of the following does Jamie have – but NOT Alex? (Stomach flu resistance)
27. Which of the following does Alex have – but NOT Jamie? (Increased Alzheimer's risk)
28. Which of the following does Alex have – but NOT Jamie? (Increased risk of esophageal cancer)
29. Which of the following does Alex have – but NOT Jamie? (Reduced risk of age-related macular degeneration)
30. Which of the following does Alex have – but NOT Jamie? (Reduced risk of obesity)
31. Which of the following does Alex have – but NOT Jamie? (Stomach flu resistance)
32. The information in the report is presented in a clear and accessible manner.
33. The overview report is easy to understand.
34. The overview report is easy to explore.
35. I would need the help of a healthcare professional to better understand these results.
36. The report gives me a firm grasp of Jamie's health and genetics.
37. The report gives me a firm grasp of Alex's health and genetics.
38. The visualization communicates health concerns in a clear way.

39. I am able to grasp to what extent Jamie and Alex share health concerns.
40. I found the ability to filter helpful for interacting with this report.
41. Please use the space below to tell us which features were most helpful for understanding and comparing the reports.
42. Please use the space below to tell us how we can improve this tool to make it easier to understand and compare reports.
