# Supplementary material for: Exploring Genetic Data Across Individuals: Design and Evaluation of a Novel Comparative Report Tool
Source: J Med Internet Res. 2018 Sep 24;20(9):e10297. doi: 10.2196/10297 (PMC6231826; doi:10.2196/10297)
Supplement: Multimedia Appendix 2 [file jmir_v20i9e10297_app2.pdf]

Appendix 2: User Study 2 Questionnaire

1. How old are you?
2. What is your gender?
3. Level of education completed:
4. Do you work in the life sciences?
5. Did you study life sciences at a collegiate or higher level?
6. Have you had your genome mapped before?
7. Which genetic testing service did you use?
8. What reports or tools did you use to view and learn from your personal genomic data?
9. Which pathogenic variants (both potentially affected and carrier) are shared by all the family members (both children and both parents)?
10. Which pathogenic variants (both potentially affected and carrier) does Parent 2 share with at least one child?
11. Which of the following family member(s) do you expect to not be resistant to norovirus (stomach flu)? Check all that apply:
12. Which of the following family member(s) has a gene variant indicating they may have a lower risk for breast cancer? Check all that apply:
13. Which of the following family member(s) has a gene variant indication they may have drug-induced arrhythmia? Check all that apply:
14. Which child shares the most cancer-related variants with Parent 1?
15. Parent 1 has _____ cancer-related variants than Parent 2.
16. Which variants are not expected to affect Child 1 themselves, but may affect Child 1's future children?
17. Which variant would Child 1 be most likely to discuss with a healthcare provider, and why?
18. Which variant would Child 2 be most likely to discuss with a healthcare provider, and why?

19. Which variant would Parent 1 be most likely to discuss with a healthcare provider, and why?
20. Which variant would Parent 2 be most likely to discuss with a healthcare provider, and why?
21. Which of the following family members have increased risk of of Alzheimer's? Check all that apply:
22. Which of the following family members have reduced risk of getting the stomach flu? Check all that apply:
23. The information in the report is presented in a clear and accessible manner.
24. The overview report is easy to understand.
25. The overview report is easy to explore.
26. I would need the help of a healthcare professional to better understand these results.
27. The report gives me a firm grasp of Child 1's health and genetics.
28. The report gives me a firm grasp of Child 2's health and genetics
29. The report gives me a firm grasp of Parent 1's health and genetics.
30. The report gives me a firm grasp of Parent 2's health and genetics.
31. The visualization communicates health concerns in a clear way
32. I am able to grasp to what extent Child 1 and Child 2 share health concerns.
33. I found the ability to filter helpful for interacting with this report.
34. Please use the space below to tell us which features were most helpful for understanding and comparing the reports.
35. Please use the space below to tell us how we can improve this tool to make it easier to understand and compare reports.
