# Supplementary material for: Exploring Genetic Data Across Individuals: Design and Evaluation of a Novel Comparative Report Tool
Source: J Med Internet Res. 2018 Sep 24;20(9):e10297. doi: 10.2196/10297 (PMC6231826; doi:10.2196/10297)
Supplement: Multimedia Appendix 3 [file jmir_v20i9e10297_app3.pdf]

Appendix 3: User Study 3 Questionnaire

1. What are your reasons for exploring information about your genetics? Please select the personal importance of each of the following reasons: [To learn personal disease risk or health-related information] [Curiosity] [To contribute to research] [Interested in distant ancestry (race/ethnicity)] [Learning more about my family origin and recent ancestry] [To provide disease risk information for children and other family members] [To learn more about myself] [Understanding this data for professional purposes] [Other]
2. If you selected "Other" above, please specify:
3. When did you receive your genome or genetic testing results?
4. What reports or tools did you use to view and learn from your personal genomic data previous to this visualization?
5. What new insights and information about your genetics did this visualization give you?
6. Please list the gene variants you found most interesting and describe how and why you identified them as interesting.
7. If you used the comparison tool, please list to whom you compared your data and what you learned from this comparison.
8. Are there any other potential people (e.g. parent, sibling, spouse, children) to whom you'd like to compare your data? If so, what questions would you like to explore in comparison to these individuals?
9. Please use the space below to tell us which features were most helpful for understanding the report and why they were helpful.
10. Please use the space below to tell us how we can improve the report to make it easier to understand.
11. Please elaborate on anything new you learned from this visual report that you didn’t notice in previous reports.
12. Please tell us more about your interaction with the report: [The information in the report is presented in a clear and accessible manner.] [I would need the help

of a healthcare professional to better understand my results] [The report gives me a firm grasp of my health and genetics] [Using this visualization I learned new insights and information about my genetics that I hadn’t noticed in previous reports] [I am able to grasp to what extent the knowledge regarding different variants is certain or uncertain] [I found the full glossary helpful for interacting with my report] [I found the ability to save variants helpful for interacting with my report] [The visualization changed my understanding of my report] [I learned something new from comparing my data to another person]

13. What is your age?
14. What is your gender?
15. Level of education completed:
16. Do you work in the life sciences?
17. Did you study life sciences at a collegiate or higher level?
18. Please use the space below for any additional thoughts that haven't been addressed in our previous questions. Thank you for your feedback!
